# Caffeine gum improves 5 km running performance in recreational runners completing parkrun events

**DOI:** 10.1007/s00394-024-03349-3

**Published:** 2024-02-24

**Authors:** Anthony Lynn, Chloe Shaw, Anna C. Sorsby, Pippa Ashworth, Faysal Hanif, Claire E. Williams, Mayur K. Ranchordas

**Affiliations:** 1 Food Group, Department of Service Sector Management, College of Business, Technology and Engineering, Sheffield Hallam University, Sheffield, S1 1WB UK; 2 Advanced Wellbeing Research Centre, Olympic Legacy Park, 2 Old Hall Road, Sheffield, Sheffield, S9 3TU UK; 3 Academy of Sport and Physical Activity, Sheffield Hallam University, Sheffield, S10 2BP UK; 4 Molecular Gastroenterology Research Group, Department of Oncology and Metabolism, The Medical School, The University of Sheffield, Beech Hill Road, Sheffield, S10 2RX UK

**Keywords:** Caffeine gum, Recreational runners, Running events, Parkrun

## Abstract

**Purpose:**

The purpose of this study was to determine whether caffeine gum improves the performance of recreational runners completing parkruns (weekly, 5 km, mass participant running events).

**Methods:**

Thirty-six recreational runners (M = 31, F = 5; age 33.7 ± 10.7 y; BMI 23.1 ± 2.4 kg/m^2^) capable of running 5 km in < 25 min were recruited to a study at the Sheffield Hallam parkrun, UK. Runners were block randomized into one of three double-blind, placebo-controlled, cross-over intervention trials with caffeine gum as the treatment (*n* = 6 per intervention trial) or into one of three non-intervention trials that ran concurrently with the intervention trials (*n* = 6 per non-intervention trial). Changes in conditions across different parkruns were adjusted for using data from the non-intervention trials. Runners in the randomized cross-over intervention trials chewed gum supplying 300 mg of caffeine or a placebo gum for 5 min, starting 30 min before each parkrun.

**Results:**

Caffeine gum improved 5 km parkrun performance by a mean of 17.28 s (95% CI 4.19, 30.37; *P* = 0.01). Adjustment for environmental conditions using data from the non-intervention trials attenuated the statistical significance (*P* = 0.04). Caffeine gum also decreased RPE by 1.21 (95% CI 0.30, 2.13; *P* = 0·01) units relative to placebo.

**Conclusions:**

A 300 mg dose of caffeine supplied in chewing gum improved the performance of recreational runners completing 5 km parkruns by an average of 17 s.

**Trial Registration:**

The study was registered at ClinicalTrials.gov: NCT02473575 before recruitment commenced.

## Introduction

Caffeine is widely used by elite athletes to improve performance. An investigation into the prevalence of caffeine use amongst elite athletes found that 74% of Olympians used some form of caffeine supplement during the 2004–2008 Olympic cycle [1]. Data on caffeine use among recreational athletes including runners are lacking, however, numerous articles on the websites of running magazines extol the benefits of caffeine for enhancing running performance [2, 3]. Thus, it seems probable that many recreational runners may consume caffeine before or during a run with the aim of improving their performance. The mechanisms through which caffeine could enhance running performance have not been definitively determined but probably include effects on neural transmission, arousal, and pain perception mediated through caffeine’s ability to act as an adenosine receptor antagonist [4].

A meta-analysis of 44 studies on caffeine and endurance performance reported that moderate doses of caffeine (3–6 mg·kg^−1^) caused a small 2.3% reduction in time trial completion time [5]. However, most of the studies included in the meta-analysis were laboratory based and it is uncertain whether laboratory protocols translate to improved performance in real world running events. To our knowledge, no study has investigated the effect of caffeine supplementation on the performance of recreational runners taking part in mass participation running events. Several studies have, however, investigated the effect of caffeine supplementation on endurance running performance using field study/race simulation protocols, albeit, with predominately well-trained runners. Bridge & Jones [6] reported that ingestion of 3 mg·kg^−1^ of caffeine enhanced performance by 1.2% (95% CI 0.7, 1.8) in eight trained male distance runners competing against each other in 8 km races on an outdoor running track. Similarly, O’Rourke et al. [7] observed that an intake of 5 mg·kg^−1^ of caffeine before a 5 km running time trial on an outdoor track improved the performance of 15 recreational runners by 1.0% (95% 0.2, 2.0) and 15 trained runners by 1.1% (95% CI 0.4, 1.6). Clarke et al. [8]demonstrated that drinking coffee (supplying approximately 3 mg·kg^−1^ of caffeine) 60 min before a 1 mile running race on an indoor track improved performance of 13 trained male runners by 1.3% compared with decaffeinated coffee and 1.9% compared with placebo. Whalley et al. [9] reported that caffeine (3–4.5 mg·kg^−1^) supplied in tablets 15 min before a 5 km outdoor, self-paced, time trial caused a statistically significant 2.0% ± 1.1 improvement in running performance relative to placebo. However, supplying the same dose of caffeine in strips or gum produced smaller increases in performance of 1.2% ± 1.0 and 0.9% ± 1.4, respectively, neither of which were statistically different to placebo. Cohen et al. [10] found that ingestion of 5 mg·kg^−1^ and 9 mg·kg^−1^ of caffeine failed to improve performance in 7 trained endurance runners (5 males and 2 females) completing 21 km field trials on a road course in hot and humid conditions. Collectively, these studies provide reasonably consistent evidence that caffeine supplementation may improve running performance by 1% to 2% in simulated races, especially in trained runners. Whether this ergogenic effect is robust for recreational runners taking part in real world running events where changing weather conditions, differences in the field of runners/competition and other external motivating factors [11] may influence performance requires confirmation.

In performance studies, caffeine has normally been administered in capsules or dissolved in a drink and taken 60 min before exercise [5]. This pre-exercise timing is commonly used because it typically coincides with peak blood caffeine concentration after an oral dose [12]. Caffeine gum provides an alternative vehicle for supplying caffeine. Possible advantages of ingesting caffeine in chewing gum include a more rapid initial absorption [13] and, therefore, onset of action [13, 14] and possibly less gastrointestinal discomfort because the absorption of a substantial proportion of the caffeine dose has been purported to occur in the mouth [13]. A decrease in the risk of gastrointestinal discomfort could make caffeine gum an attractive supplement for any runners who experience gastric distress when running after consumption of caffeine. Supplementation with gum supplying 200–300 mg of caffeine has been shown to enhance repeated sprint cycling [15], time-trial cycling [16], countermovement jump height [17, 18], and intermittent running [18]. However, as previously noted, Whalley et al. [9] found only a small non-significant improvement in running performance when caffeine gum (200 mg caffeine for runners < 65 kg and 300 mg for runners > 65 kg) was administered 15 min before a 5 km run. They subsequently attributed the limited effect of the gum to providing it too long before start of the run [19].

The aim of the present study was to determine the effects of caffeine gum on the performance of recreational runners taking part in mass participation running events. We used parkrun events to conduct the study. Parkruns are free, weekly, timed, 5 km running events that originated in the UK, but now occur in locations around the globe [20]. Parkruns attract predominately recreational runners, some of whom may conceivably use caffeine to enhance their performance. As such, findings from this study could inform the pre-event nutritional practices of recreational runners taking part in parkruns or similar popular mass participation running events.

## Materials and methods

### Experimental design

The overall study contained three caffeine intervention trials and three non-intervention trials. The three caffeine trials compared caffeine gum versus placebo using a double-blind, block randomized, cross-over, study design. The three non-intervention trials consisted of runners that completed two 5-km parkruns with no intervention. The inclusion of the non-intervention trials was at the behest of the parkrun Research Review Board who expressed concern that changes in weather and other variables such as the total number of runners between parkruns could mask or inflate the effect of caffeine supplementation. Data from the non-intervention trials were used to account for the impact of changes in such variables between parkruns on the performance of runners in each cross-over caffeine trial (see ‘Statistical Analysis’ section for further details). Participants were assigned to the caffeine gum trials or non-intervention trials using block randomization (See Protocol/Controls for further details). Those assigned to the caffeine trials then completed a cross-over study of caffeine chewing gum versus placebo gum. Three cross-over trials with 6 runners in each trial were conducted. The non-intervention trials ran concurrently and contained 6 runners per trial. So, at any single parkrun, data were collected from a maximum of 12 runners. For trial one, the two 5-km parkruns were separated by 21 days, whereas for trials two and three, the parkruns were separated by 7 days. The longer time lapse between the two parkruns in trial one was because the weekend after the first parkrun there was a special parkrun that did not follow the normal course route and the following week, the parkrun was cancelled to accommodate a public event in the park. Each parkrun commenced at 9 am on a Saturday morning and was held at Endcliffe Park, Sheffield, UK. The environmental conditions on each run day are reported in Table 1.

**Table 1:** Environmental conditions on each study day

Trial	Week	Temperature (°C)	Humidity %	Wind speed (MPH)
1	1	15.7	77	5
	2	16.0	74	6
2	1	10.1	87	3
	2	7.5	94	2
3	1	6.0	95	11
	2	3.0	73	14

Participants in the caffeine gum and non-intervention trials were instructed to avoid caffeine from 3 pm on the day before each 5 km run and to not undertake any strenuous exercise for 48 h before each run. To check compliance with these instructions, participants were asked to complete a diet dairy for 24 h before each run and a physical activity dairy for 48 h before each run. Inspection of the diaries revealed that all runners complied with the instruction to avoid caffeine and to minimize strenuous physical activity. Participants were instructed to follow their normal morning routines for each parkrun including eating the same foods and wearing the same running shoes. Sheffield Hallam parkrun attracts over 400 runners, so participants were instructed to start from the same location within the bunch of runners each week. At the end of each run, participants provided ratings of perceived exertion (RPE) and those in the intervention trials were asked whether they thought they had received caffeine gum or placebo gum.

### Participants

A total of 36 recreationally trained runners were recruited. For this study, participants were classed as recreational runners if they took part in mass participation running events but did not compete in elite level races. All data collection took place at Endcliffe Park, Sheffield, UK between July 2015, and January 2016. Inclusion criteria were aged 18–65 y, capable of running 5 km in < 25 min, recent completion of at least one parkrun, no previous adverse responses to caffeine, no muscle injuries, and free of known disease. Being able to run 5 km in < 25 min was used as an inclusion criterion, because finishing time data on the parkrun website indicated that there was a larger between run variation in runners completing parkrun in > 25 min than in those completing runs in < 25 min. Participants were recruited by word of mouth and advertisement at Sheffield Hallam and Hillsborough parkruns, Sheffield, UK. All participants were habitual consumers of caffeine.

### Sample size

The pre-specified primary outcome variable was change in 5 km finishing time in the runners randomized to the three caffeine trials. Sample size for statistical significance was calculated using an online sample size calculator [21] and was based on an expected improvement of 1.2% in running times [6] and a within-runner variation of 1.26% (calculated from data on weekly parkrun times of runners completing Sheffield Hallam parkrun in < 25 min in spring 2014 [22]. Considering a power of 80% and an alpha value of 0.05, a total sample size of 19 was calculated for the caffeine trials. Recruitment of runners to the caffeine trials was halted at 18 to finish the study within the time restriction set by the parkrun organization. The same number of runners were recruited to the non-intervention trials. Pre-planned secondary outcome variables were the effect of caffeine gum on RPE, pacing (determined from 1 km split times) and heart rate.

### Interventions

Participants randomized to the caffeine trials consumed either three pieces of caffeine gum (Military Energy Gum, Marketright Inc., USA) or three pieces of placebo gum, thirty minutes before each parkrun. The three pieces of gum were chewed concurrently for 5 min. Chewing time was monitored by the research team to ensure that all participants chewed their gum for the same duration. Expectorated gum was collected into plastic bags for disposal. Both gums were matched for taste (Artic mint) and appearance (bright blue). The caffeine and placebo gum were provided by the manufacturer free of charge. According to the manufacturer, each piece of caffeine gum contained 100 mg of caffeine. We analyzed the caffeine content of the gum using high performance liquid chromatography (HPLC) and found a mean caffeine content of 92 (range 90.5–93.5) mg per piece of gum. Our caffeine analysis method is reported elsewhere [23]. The gum was covered by a hard-shell coating that hindered the preparation of samples for HPLC analysis and this may explain the slightly lower value we found relative to the claims of the manufacturer. The ingredients and nutritional composition of the caffeine and placebo gums are shown in Table 2.

**Table 2:** Macronutrient composition and ingredients of caffeine gum*

Macronutrient	Per piece
Energy (kcal)	10
Carbohydrate (g)	2
Fat (g)	0
Protein (g)	0
Caffeine according to product label (mg)	100
Analysed caffeine content (mg)	92 (range 90.5–93.5)

Ingredients: Sugar, Dextrose, Gum Base, Natural and Artificial Flavors, Caffeine, Glycerine, Corn Syrup, Aspartame-Acesulfame, Maltodextrin, Sucralose, Aspartame, Artificial Colours (including Blue 1 Lake) Resinous Glaze, Carnauba Wax, Neotame, Soy Lecithin, and BHT

*The placebo gum contained the same ingredients and macronutrient composition as the caffeine gum, except that it was devoid of caffeine

### Measurements

Finishing time and split times for each 1 km were recorded manually using Geonaute On Start 700 stopwatches. All runners were fitted with a Garmin Forerunner 20 GPS/HR monitor watch to record heart rate. The faces of the watches were covered with an adhesive strip so that runners could not see the time. Average RPE for each run were collected at the end of each run using a 6–20 Borg scale [24]. Self-reported height and mass of each runner were collected.

We used parkrun as our test protocol for caffeine supplementation because we wished to study the ergogenic effect of caffeine gum during a real-world mass participation running event so our results would be ecologically valid, and of direct relevance to recreational runners. An advantage of using parkruns was they occurred weekly, so it was possible to have a brief time lapse between repeated runs to minimize the effects of changes in fitness. Moreover, data from previous events were available to estimate the within-person SD to inform a sample size calculation.

### Protocol/controls

Block randomization sequences (block size 6) were constructed as described in Altman [25] to allocate runners to the caffeine trials or non-intervention trials and to determine supplementation order in the caffeine trials [25]. The order of blocks was chosen at random using an online random number generator (Random.Org) [26] by a colleague not involved in data collection. Placebo and caffeine gums were provided to researchers in identical plastic bags labelled with the participants’ ID numbers. So, researchers and participants were blinded to treatment allocation for the duration of the data collection.

### Statistical analysis

Data from the three caffeine trials were combined. The effects of the caffeine gum on 5 km performance (primary outcome variable) and RPE were assessed using paired samples t-tests. Sequence effects for performance and RPE were assessed by comparing the effect of caffeine gum in those participants allocated caffeine first versus those allocated placebo first, using independent samples *t*-tests. A secondary statistical analysis explored the possible effect of changing conditions at each run, on finishing time. The time of individual runners in each of the three cross-over caffeine trials was divided by the mean time of the group of runners in the associated non-intervention trial to produce a ratio. These ratio data were then analyzed using a paired samples t-test. For all paired samples t-tests, the paired differences between treatments were normally distributed as determined by Shapiro–Wilk tests. For the independent samples t-tests the data was normally distributed (Shapiro–Wilk test) and there was homogeneity of variance (Levene’s test).

Pearson’s r correlation was used in exploratory analyses to investigate the relationship between the magnitude of performance enhancement by caffeine and: (i) running ability, (ii) self-reported body mass and (iii) age.

Problems with the manual timing of the 3 km distance split in trial two meant that complete km time splits for participants were only available for trials 1 and 3. A repeated measures ANOVA (with treatment and km split times as within group factors) was used to investigate the effect of caffeine gum on overall pacing using the combined data from trials 1 and 3 (*n* = 10). The data and residuals passed tests of sphericity and normality. Because of the failure to capture the 3 km split times from trial 2, a further unplanned exploratory analysis was conducted to explore whether runners started their parkruns proportionally faster after receiving caffeine gum than after placebo gum. Using data from all three intervention trials (*n* = 14) the proportion of the overall finishing time of each runner accounted for by the time of the first 1 km was calculated by dividing each runner’s first 1 km split by their finishing time. The resulting ratios were then analyzed using a paired samples *t*-test. The paired differences of these ratios passed the Shapiro–Wilk test for normality.

All statistical analyses were conducted on SPSS version 24.0 (IBM, 2016).

## Results

### Participant characteristics and retention

The characteristics of the runners are shown in Table 3. Of the 36 runners recruited, 29 completed the study, 14 from the caffeine intervention trials and 15 from the non-intervention trials. Of the 7 runners that withdrew from the study, all failed to attend their second run; 3 withdrew because of unexpected work commitments (1 caffeine; 2 non-intervention), 2 because they suffered an injury between the 2 runs (1 caffeine; 1 non-intervention), 1 because they attended a party the night before their scheduled run (caffeine), and 1 provided no reason (caffeine).

**Table 3:** Characteristics of participants in each study at enrolment (mean, SD)

Trial	Group	Age (y)	Sex (M/F)	Height (m)	Mass (kg)	BMI (kg/m^2^) *
1	Intervention	38.0 (13.11)	5/1	1.80 (0.06)	78.0 (10.20)	23.9 (1.97)
	Non-intervention	29.3 (7.28)	5/1	1.75 (0.05)	73.0 (8.37)	23.8 (2.12)
2	Intervention	24.0 (9.42)	6/0	1.81 (0.09)	73.7 (6.79)	22.6 (1.57)
	Non-intervention	36.5 (10.21)	5/1	1.69 (0.05)	64.6 (11.00)	22.4 (2.75)
3	Intervention	33.0 (9.88)	6/0	1.78 (0.10)	77.1 (10.76)	24.4 (2.28)
	Non-Intervention	37.2 (10.61)	4/2	1.76 (0.09)	74.2 (18.44)	23.6 (3.46)

*Calculated from self-reported height and mass

### Effects on 5 km run performance

**Fig. 1:**
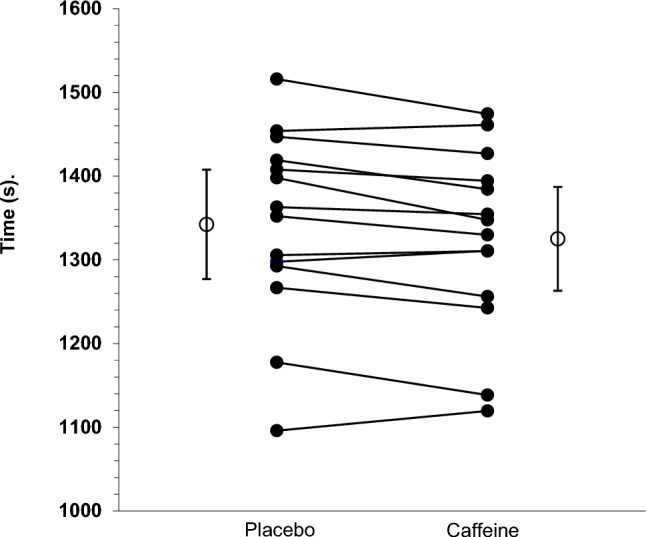
Effect of caffeine gum on 5 km finishing time (*n* = 14). Error bars associated with group means are 95% CI

The primary statistical analysis using data from the caffeine trials revealed that caffeine gum reduced parkrun finishing time by 17.28 s (95% CI 4.19, 30.37; *t* (13) =  – 2.85, *P* = 0.01) (Fig. 1.). Inspection of individual responses to the caffeine gum showed that performance improved in 10 of the 14 runners, but the magnitude of the improvement was variable (Fig. 1). There was no statistically significant effect of supplementation order on the effects of caffeine gum (*t* (12) =  – 1.182, *P* = 0.26). There was no statistically significant correlation between placebo running time (crude proxy of training status) and the percentage improvement in performance observed after caffeine gum (*r* = 0.31; *P* = 0.25). Despite all runners receiving a fixed dose of caffeine (300 mg) irrespective of their body mass, we found no significant correlation between self-reported body mass and the magnitude of improvement in performance elicited by the caffeine gum (*r* = 0.11; *P* = 0.71). There was also no significant correlation between the age of the runners and the improvement in performance caused by caffeine gum (*r* = 0.15; *P* = 0.61).

A secondary analysis to investigate the effect of changing conditions between parkruns on the ergogenic effects of caffeine gum incorporated the finishing time data of the groups of runners from the non-intervention trials into the statistical analysis. The effects of caffeine gum on parkrun performance remained statistically significant in this analysis, but the statistical significance was reduced (Mean difference in ratio  – 0.014, 95% CI  – 0.027,  – 0.001; *t* (13) =  – 2.32, *P* = 0.04).

### Effects on RPE and heart rate

**Fig. 2:**
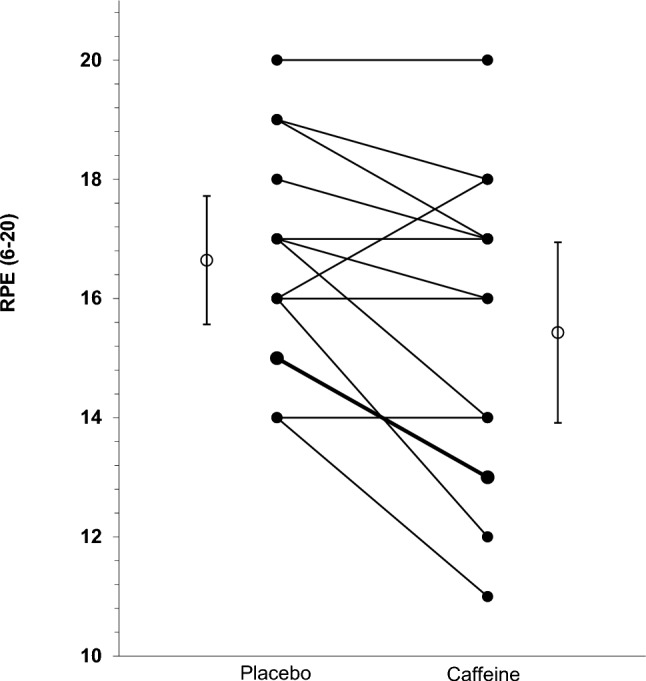
Effect of caffeine gum on RPE (6–20 Borg scale; *n* = 14). Error bars associated with group means are 95% CI. Thicker line represents two participants with the same RPE values for placebo and caffeine

RPE at the end of each parkrun was significantly lower after caffeine gum than placebo gum (15.43 v 16.64, mean difference  – 1.21 95% CI  – 0.30,  – 2.13; *P* = 0.01). There was no statistically significant effect of gum order on RPE (*t* (13) = 0.837; *P* = 0.42). Inspection of the data revealed that 9 runners reported a reduced RPE after caffeine, 4 reported no change and 1 reported an increase (Fig. 2). Technical issues with the heart rate monitors meant there was insufficient data to investigate the effects of caffeine gum on heart rate.

### Effects on pacing and km splits times

An analysis of 10 participants for which complete km split times were collected revealed no evidence of an effect of caffeine gum on pacing (treatment x time interaction F (4) = 1.54; *P* = 0.21). An exploratory analysis comparing the ratio of the first km split times to 5 km finishing times for all 14 runners in the caffeine intervention trials found no statistically significant difference between caffeine gum and placebo (mean difference in ratio  – 0.005, 95% CI  – 0.0099, 0.0003; *t* (13) =  – 0.20, *P* = 0.06).

### Side effects and treatment allocation

There were no reports of any adverse effects from chewing the caffeine gum. The 14 runners who completed the caffeine trials were asked to identify which gum they received at the end of their first and second runs. At the end of their first run, 5 of 14 runners correctly identified which gum they had received (3 on caffeine and 2 on placebo). At the end of their second run, this rose to eight of 14 runners (3 on caffeine and 5 on placebo).

## Discussion

To our knowledge, this is the first study to investigate the effect of caffeine on the performance of recreational runners completing mass participation running events. The main finding was that 300 mg of caffeine delivered in chewing gum enhanced the performance of runners completing 5 km parkruns by 17 s, which equates to an improvement of 1.3%. This is comparable with the 1.0–2.0% improvements observed in field studies and race simulations of 5 to 8 km distance after supplementation with 3 to 5 mg·kg^−1^ of caffeine [6, 7, 9]. Caffeine gum also reduced reported RPE at the end of each parkun.

When we analyzed the data from the intervention trials, we found clear evidence of an ergogenic effect of caffeine gum on parkrun performance. However, because we were collecting data from outdoor parkrun events where weather conditions and the field of runners could vary unpredictably between parkruns; we conducted a secondary statistical analysis that attempted to account for the influence of these factors. To do this, we collected data from groups of runners who ran the same parkruns as our caffeine intervention runners but did not receive an intervention. Changes in their running times were then used to adjust the running performance of the intervention runners (see ‘Statistical Analysis’ section of the methods for more details). In this secondary analysis, the statistical significance was reduced indicating that uncontrollable factors that differed between the parkrun events such as change in weather conditions and field of runners may have contributed to the change in performance of the runners in the caffeine intervention. However, it is noteworthy that improvements in performance were statistically significant in both analyses. As such we believe we found reasonably robust evidence that caffeine gum benefitted the performance of recreational runners completing 5 km parkrun events.

To our knowledge, only one other study has reported on the effect of caffeine gum on 5 km running performance [9]. In that study, Whalley et al. [9] compared the effect of caffeine (200 mg for runners < 65 kg and 300 mg for runners > 65 kg) in gum with the same dose supplied in tablets and mouth strips. Only caffeine tablets significantly improved performance relative to placebo (2.0% ± 1.1). When Whalley et al. [19] subsequently compared the ratio of caffeine to paraxanthine in urine samples collected at the end of the 5 km run they observed a higher ratio after consumption of caffeine tablets than after caffeine gum or oral strips. This indicated that caffeine from the gum and oral strips was metabolised more quickly than from tablets and led Whalley et al. [19] to propose that caffeine gum may need to be administered closer than 15 min before the start of an event to exert its maximal effect. In support of their proposition, a recent meta-analysis reported that caffeine gum was ergogenic when supplied < 15 min before the start of exercise but not when supplied > 15 min before [27]. However, the meta-analysis contained only three studies in the > 15 min grouping, and the estimate of effect had a wide CI so the authors concluded that their result had low certainty and should be interpreted cautiously.

The results of our study disagree with the proposition that caffeine gum needs to be supplied < 15 min before exercise to exert a significant effect because we administered the gum 30 min before exercise commenced. A pharmacokinetic study we conducted after data collection for the current study was completed may provide some insight into why we found an ergogenic effect despite giving caffeine gum 30 min before the start of the parkruns. In that study, we observed that when one piece of caffeine gum was chewed, a first peak in blood caffeine concentration occurred at approximately 10 min, but this was followed by a second substantial, albeit variable peak, at approximately 45 min in most individuals, which we ascribed to caffeine being swallowed in the saliva and then absorbed more distally in the gastrointestinal tract [23]. So, it is possible that the runners in our study experienced a second increase in caffeine in their bloodstream during their 5 km run, whereas in the study of Whalley et al. [9] runners would have finished their runs before this second peak occurred. However, further studies are needed to clarify the optimal timing of caffeine gum use before exercise.

Although we found an improvement in mean 5 km running time after caffeine gum, there was considerable interindividual variability in response, with 4 participants running more slowly after caffeine gum. Interindividual variation in response to caffeine has been frequently reported and attributed to various factors such as differences in performance ability [28], dose of caffeine, and single nucleotide polymorphisms (SNPs) in genes involved in caffeine metabolism (CYP1A2) [29] or sensitivity to caffeine (adenosine A2a receptor gene; ADORA2a) [30]. Our study was not designed nor powered to explore factors that might influence individual responses to caffeine. Nevertheless, we conducted exploratory analyses to determine whether the magnitude of the ergogenic effect of caffeine correlated with placebo running performance (used as a proxy measure of performance ability), dose of caffeine and age. We only found a small non-significant correlation between the magnitude of enhancement of performance with caffeine supplementation and placebo running performance, which contrasts with a previous study that found a strong inverse correlation [9]. The relative exposure to caffeine for each runner varied because we supplied a fixed 300 mg dose of caffeine. So, we explored whether the magnitude of improvement in performance correlated with body mass but did not find a significant correlation. This lack of correlation needs to be interpreted with caution because it was based on self-reported body mass. There is limited and equivocal evidence on whether the ergogenic effect of caffeine changes with age [31, 32], but because our runners varied in age from 18 to 62, we investigated whether age correlated with caffeine induced increases in performance. However, we only found a small non-significant correlation suggesting that age was not a major determinant of the extent to which caffeine gum improved parkrun running performance.

A secondary aim of the present study was to explore the effect of caffeine gum on pacing during the parkrun, but a timing error in trial 2 meant that km split times for each km were only available for trials 1 and 3. An analysis restricted to the runners from these two trials failed to identify a statistically significant interaction between caffeine gum and pacing. Since we had complete data for the first 1 km splits for runners from all three trials, we conducted an unplanned exploratory analysis to determine whether runners started their parkrun proportionally faster after caffeine consumption, but the difference between caffeine and placebo was not statistically significant. Our failure to detect an effect of caffeine supplementation on pacing contrasts with Santos et al. [33] who reported that caffeine supplementation (5 mg·kg^−1^) altered pacing strategy in a 4 km cycling time trial, with effects on pacing becoming evident after approximately 1.5 min [33]. The disagreement between our study and Santos et al. [33] could reflect differences in the exercise protocol; a 4 km cycle time trial lasting a mean of just under 7 min versus a 5 km run lasting a mean of approximately 22 min. The runners in our study also received a lower dose of caffeine. We supplied caffeine gum that contained approximately 300 mg of caffeine, but it is likely that our 5-min chewing protocol only released a mean of 77% of this caffeine i.e. 231 mg [23]. Thus, it seems our runners were exposed to between 2.48 and 3.79 mg·kg^−1^ of caffeine. It is possible that a higher dose may be needed to substantially alter pacing.

Caffeine caused a mean 1.21 units reduction in RPE measured at the end of the parkrun. Inspection of individual participant data indicated that RPE was reduced or unchanged for all but one runner. This suggests that caffeine gum dampened perceptions of effort during the parkrun and this may have contributed to the performance enhancing effect we observed. Evidence tends to support the concept that caffeine increases the workload to RPE ratio during performance tests but that does not always translate to a reduction in RPE [28, 34]. For example, Astorino et al. [28] reported that caffeine supplementation (5 mg·kg^−1^) enhanced 10 km cycle time trial performance without changing RPE [28] and Bridge & Jones [6] found that caffeine (5 mg·kg^−1^) improved 8 km running performance, but only caused a non-significant trend towards a lower RPE.

The major strength of the current study was that we evaluated the effect of caffeine gum in a 5 km mass participation running event, so our results are of direct relevance to recreational runners of a comparable fitness level completing mass participation events of a similar distance. We also believe the performance effects we observed were unlikely to represent a placebo effect, because participants struggled to correctly identify which supplement, they had taken after each run. Nervousness associated with completing a parkrun and other distractions related to the events may have helped to mask the arousal effects of the caffeine gum that typically make successful blinding of participants in caffeine studies very difficult [11].

This study has several limitations, some of which exemplify the difficulty of conducting an intervention study using mass participation running events. First, we experienced a high dropout rate with 4 of 18 runners in the caffeine trials and 3 of 18 runners in the non-intervention trials failing to complete their two parkruns, mainly because of unexpected work and social commitments. Ideally, we would have conducted a further trial to account for dropouts and to meet our pre-planned sample size calculation, but this was not possible because the duration of permission from the parkrun organization to conduct the study expired. Second, a timing problem during the second trial meant no times were recorded for the 3 km split; this limited our ability to investigate the overall effect of caffeine on pacing. Third, some heart rate monitors failed to record data throughout the whole of each parkrun, so it was impossible to accurately determine the effect of caffeine gum on heart rate. Fourth, we asked participants to chew the gum for 5 min based on replicating chewing protocols from the literature [15, 16, 35] at the time we designed the study, but since then, we have demonstrated that a chewing time of 5 min extracts only 77% (range 67 – 86%) of the caffeine from gum whereas prolonging the chewing time to 10 min increases the amount of caffeine extracted to 96% and reduces interindividual variation [23]. So, it is possible we may have observed a greater and/or more consistent effect of caffeine gum if we had implemented a chewing duration of 10 min. Fifth, we collected self-reported body mass rather than directly measuring body mass, so the lack of a significant correlation between body mass and the magnitude of performance enhancement caused by caffeine needs to be interpreted cautiously. Sixth, we had one female runner in the caffeine intervention trials and four in the non-intervention trials. We did not control for each participant’s menstrual cycle, and this may have impacted on their parkrun performance and the results of this study. Finally, the public event setting meant that it was not feasible to collect pre- and post-run blood samples. These could have provided useful information on the extent of inter-individual variation in the elevation of plasma caffeine concentration after the consumption of caffeine gum and may have explained some of the between person variation in response we observed.

In conclusion, 300 mg of caffeine supplied in chewing gum decreased 5 km parkrun time by a mean of 17 s. This may be of interest to recreational runners taking part in similar events given the lack of reported adverse effects and ease with which caffeine gum can be incorporated into preparation routines. Future studies need to identify the optimal dose, chewing duration, and timing of administration of caffeine gum to maximize its ergogenic potential, whilst minimizing the risk of any adverse effects.
